# Impact of systemic steroid treatment on talc pleurodesis: a report of six cases

**DOI:** 10.1186/s40780-020-00171-x

**Published:** 2020-07-03

**Authors:** Soichiro Ishii, Hiroshi Sakurashita, Emiko Mashida, Yasuyuki Saeki, Hiroaki Matsuo

**Affiliations:** Department of Pharmaceutical Services, Hiroshima University Hospital, 1-2-3, Kasumi, Minami-ku, Hiroshima-shi, Hiroshima, 734-8551 Japan

**Keywords:** Sterile graded talc, Steroids, Pleurodesis, Anti-inflammatory effects

## Abstract

**Background:**

Instillation of sterile graded talc in the pleural space is performed to prevent reaccumulation of malignant pleural effusion after drainage. Talc is thought to encourage pleural adhesions as part of the repair process by provoking inflammation, suggesting that adhesions are less likely to form in patients taking corticosteroids or other drugs with anti-inflammatory effects. However, the relationship between steroid therapy and pleurodesis efficacy remains unclear.

**Case presentation:**

We report the outcomes of six patients who underwent pleurodesis at Hiroshima University Hospital while being treated with systemic steroid therapy for non-cancer-related illnesses. Talc pleurodesis was successful at the first attempt in five of the six patients. The five successful cases were receiving low-dose oral prednisolone or methyl prednisolone (range, 1–20 mg/day) at the time of pleurodesis and had serum albumin levels ranging from 2.2 to 3.0 g/dL. In contrast, the patient in whom pleurodesis was unsuccessful was receiving a higher dose of prednisolone (40 mg/day) intravenously and had a relatively low serum album level (1.7 g/dL).

**Conclusions:**

The outcome of pleurodesis may be affected by the dose and/or route of systemic steroid therapy. Further analysis with more patients will be necessary to clarify the relationship between steroid dosage and talc pleurodesis success rate.

## Background

Pleurodesis is a procedure that aims to reduce or eliminate the pleural space by inducing adhesion of the pleural layers. Pleurodesis is most often performed to prevent fluid reaccumulation after thoracic drainage of malignant pleural effusion. The 2016 Japanese Clinical Guidelines for the Management of Lung Cancer recommends continuous thoracic drainage in cases of symptomatic cancerous pleurisy with pleural effusion [1]. Pleurodesis is recommended to manage fluid accumulation and relieve symptoms after drainage, and commonly involves agents such as OK-432 (picibanil), a preparation of inactivated *Streptococcus pyogenes*, or sterile graded talc (hereafter referred to as talc), the latter of which has been covered by Japan’s National Health Insurance since December 2013.

The mechanism of action of talc has yet to be fully elucidated; however, it is thought to provoke an inflammatory response and cytokine secretion in the pleural space that induces the formation of collagen, leading to adhesion of the visceral and parietal pleurae [2–4]. Accordingly, it has been proposed that pleural adhesion may be inhibited in patients receiving treatment with anti-inflammatory drugs, such as systemic (cortico) steroid therapy (SST) [5] and that is also mentioned in the Japanese talc package insert. However, in some patients, it may be essential to continue SST therapy while undergoing pleurodesis. In the present study, we describe the outcomes of pleurodesis in six patients who underwent talc pleurodesis at our hospital while receiving SST for non-malignant disorders.

## Case presentation

This was a retrospective study of patients who were admitted to the Department of Respiratory Internal Medicine or Thoracic Surgery at Hiroshima University Hospital between December 2013 (when insurance coverage of talc pleurodesis was approved in Japan) and March 2017. Data were analyzed from a total of 71 patients who had follow-up data available for 30 days after talc pleurodesis. Information on steroid type, administration route, and dosage; number and outcome(s) of pleurodesis procedures; serum albumin level on the date closest to pleurodesis; effusion drainage volume the day before surgery (if relevant); and subsequent adverse events (e.g., fever, pain) were obtained from medical records. Successful pleurodesis was defined as one that did not require drain placement or thoracentesis in the 30 days following the procedure.

Of the 71 patients included in the study (53 men, 18 women; median age 70 years, range 38–87 years), 6 were receiving SST (other than inhaled and topical steroids) at the time of pleurodesis (Table 1). Pleurodesis was successful in 5 of the 6 SST patients (83.7%) compared with 62 of the 65 non-SST patients (95.4%). In the SST patients, only one adverse event of pain (Grade 1, as defined by the Common Terminology Criteria for Adverse Events, version 5.0) was recorded in the 30-day follow-up period. There were no fever adverse events in SST patients. In contrast, fever and pain were observed in 8/65 (12.3%) and 9/65 (13.8%), respectively, non-SST patients. Acute respiratory distress syndrome (ARDS), a serious side effect of talc usage, was not observed in any of the 71 patients.

Table 2 shows the characteristics of the six patients on SST. Serum albumin levels were recorded because they have been reported to affect the pleurodesis success rate [6]. The five patients successfully treated by pleurodesis were receiving 1–20 mg/day of oral prednisolone (PSL) or methylprednisolone (mPSL) and had serum albumin levels between 2.2 and 3.0 g/dL, whereas the patient for whom the first pleurodesis was unsuccessful was receiving 40 mg/day PSL intravenously and had a serum albumin level of 1.7 mg/dL. The clinical courses of the six SST patients are detailed below.

**Table 1:** Patient characteristics

Characteristic	Number (*N* = 71)
Sex	
Male	53
Female	18
Age (years), median (range)	70 (38–87)
SST^a^	
Yes	6
No	65

*SST* systemic steroid therapy

^a^Excludes steroid inhalants and topical drugs

**Table 2:** Characteristics of the six patients receiving SST

Case number and pleurodesis outcome	Age (y)	Sex	Disease	Steroid	Route	Dose (mg/day)	NSAIDs	Serum albumin (g/dL)	Drainage volume on the day preceding pleurodesis (mL)	Pleurodesis procedures
(1) Success	66	F	Rheumatoid arthritis	PSL	p.o.	1	Yes	2.9	230	1
(2) Success	65	M	Kidney transplant	mPSL	p.o.	4	No	2.2	140	1
(3) Success	71	M	Interstitial pneumonia	PSL	p.o.	5	No	2.4	40	1
(4) Success	72	M	Radiation pneumonitis	PSL	p.o.	5	No	3.0	1270	1
(5) Success	66	M	Interstitial pneumonia	PSL	p.o.	20	No	3.0	50	1
(6) Failure	80	M	Nivolumab-induced pneumonia	PSL	i.v.	40	No	1.7	300	2

*NSAIDs* non-steroidal anti-inflammatory drugs, *F* female, *M* male, *PSL* prednisolone, *mPSL* methylprednisolone, *p.o.* oral, *i.v.* intravenous

### Case 1 (successful)

The patient was a 66-year-old woman with lung cancer who was taking oral PSL (1 mg/day) for rheumatoid arthritis. Her medical history included hypertension, interstitial pneumonia, bronchial asthma, hyperlipidemia, and pulmonary emphysema, and her drug history included methotrexate, folic acid, amlodipine besylate, ezetimibe, rebamipide, and omeprazole (all oral). On Day 0, she underwent upper right lobectomy (total) and middle/lower right lobectomy (partial) for lung cancer, with combined chest-wall resection and reconstruction. She was started on loxoprofen sodium hydrate (180 mg/day) after surgery. Effusion drainage volume was 1655 mL on Day 0, 2270 mL on Day 1, and 230 mL on Day 4. On Day 6, talc pleurodesis was performed to manage postoperative air leakage and pleural fluid accumulation. On Day 7, only 70 mL of effusion fluid was collected and the drainage tube was removed.

### Case 2 (successful)

The patient was a 65-year-old man with lung cancer who was taking oral mPSL (4 mg/day) and the immunosuppressants tacrolimus and everolimus following a living-donor kidney transplant. His medical history included hypertension, hyperuricemia, and hyperlipidemia, and his drug history included cilnidipine, telmisartan, febuxostat, pitavastatin calcium, teprenone, rabeprazole sodium, and sulfamethoxazole/trimethoprim (all oral). On Day 0, he underwent upper right lobectomy for lung cancer and was started on oral acetaminophen (1600 mg/day) for postoperative pain. Chylous pleural effusion leaked from his chest tube and reached 140 mL by Day 5, indicating accumulation in the intrathoracic space. Talc pleurodesis was performed on Day 7, and only 80 mL fluid was collected on Day 12. The drainage tube was removed on Day 14.

### Case 3 (successful)

The patient was a 71-year-old man with lung cancer who was taking oral PSL (5 mg/day) for interstitial pneumonia. His medical history included autoimmune hepatitis and pulmonary emphysema, and his drug history included cyclosporine, sulfamethoxazole/trimethoprim, rabeprazole sodium, and eldecalcitol (all oral). On Day 0, he was admitted for cancerous pleural effusion and labored breathing. Pleural drainage was started the same day and the effusion volume decreased from 1360 mL on Day 0 to 40 mL on Day 3. Talc pleurodesis was performed on Day 5 and the drainage volume decreased further to 10 mL on Day 6. The drainage tube was removed on Day 7.

### Case 4 (successful)

The patient was a 72-year-old man with lung cancer taking oral PSL (5 mg/day) for radiation pneumonitis. His medical history included steroid diabetes and atrial fibrillation, and his drug history included sulfamethoxazole/trimethoprim, teneligliptin hydrobromide hydrate, rivaroxaban, digoxin, and febuxostat (all oral). On Day 0, he was admitted for effusion drainage to treat recurrent chylothorax following chemoradiation therapy for lung cancer. A drainage tube was inserted on the same day and yielded 1270 mL of fluid. Talc pleurodesis was performed on Day 2, and chylous pleural effusion was managed by maintaining him on a fat-restricted diet. By Day 14, only 5 mL of fluid was collected and the drainage tube was removed on Day 15.

### Case 5 (successful)

The patient was a 66-year-old man taking oral PSL (20 mg/d) for interstitial pneumonia. His medical history included non-small cell lung cancer and diabetes and his drug history included linagliptin, mitiglinide calcium hydrate, insulin glargine, oxycodone hydrochloride hydrate, and esomeprazole magnesium hydrate (all oral). He was admitted for pleural effusion on Day 0, and a drainage tube was inserted the same day. The collected effusion was tinged with blood. The volumes gradually decreased from 840 mL on Day 0 to 50 mL on Day 4. Talc pleurodesis was performed on Day 6. On Day 7, only 60 mL of drainage fluid was collected and the drainage tube was removed on the same day.

### Case 6 (unsuccessful)

**Fig. 1:**
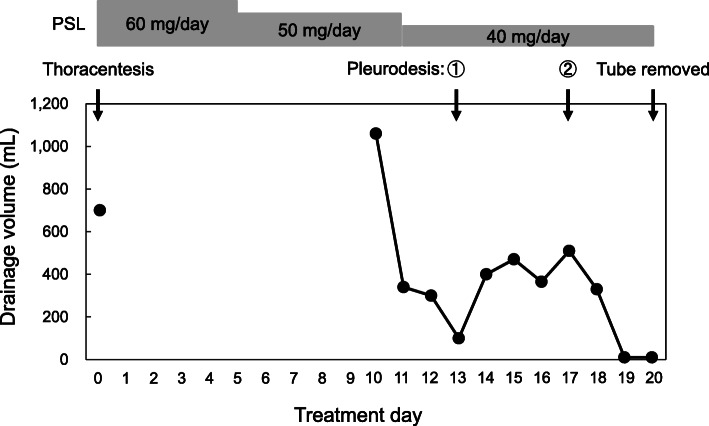
Treatment course for the case of failed pleurodesis. PSL, prednisolone

The patient was an 80-year-old man who was admitted for suspected nivolumab-induced pneumonitis and was started on intravenous PSL (60 mg/day) on the same day (Day 0). His medical history included chronic obstructive pulmonary disorder, type 2 diabetes, hyperuricemia, and carotid artery stenosis. His drug history included pioglitazone hydrochloride, alogliptin benzoate, mitiglinide calcium hydrate, insulin glargine, oxycodone hydrochloride, and esomeprazole magnesium hydrate (all oral). Thoracentesis for pleural effusion was performed on Day 0 and yielded 700 mL of fluid. Due to concerns about adverse events associated with the administration of high steroid dosages, the PSL dosage was reduced to 50 mg/day on Day 5 and to 40 mg/day on Day 11. Nevertheless, the pleural effusion gradually increased, causing respiratory discomfort, and a drainage tube was inserted on Day 10. Thereafter, the drainage volume decreased and the patient’s lungs were able to expand, allowing us to perform pleurodesis on Day 14. The patient continued to receive intravenous PSL (40 mg/day) throughout this period. Because the drainage volume remained high, pleurodesis was repeated on Day 17. The drainage volume decreased thereafter and the drainage tube was removed on Day 20 (Fig. 1). Recurrent effusion was observed on Day 29, and thoracentesis and drainage tube placement were repeated. However, the patient’s condition continued to deteriorate and he died on Day 30.

## Discussion and conclusions

This study analyzed the characteristics and outcomes of six patients who underwent talc pleurodesis while on SST. The first-time pleurodesis success rate was 83.3% (*n* = 5/6), which was roughly comparable to success rate for the patients not on SST (62/65, 95.6%). The one SST patient for whom pleurodesis was unsuccessful was taking a higher steroid dose and had a lower serum album level than the remaining five SST patients. The success of pleurodesis is reportedly lower in patients with poor general condition and low serum albumin level [6], which is consistent with our finding here. Despite the small sample number, our result suggests that temporary cessation of SST should be considered prior to pleurodesis, albeit with a tapering-off period for patients on very high steroid doses, as described for Case 6 here.

The mechanism of action of talc in pleurodesis has been widely investigated. Our current understanding suggests that pleural adhesion is stimulated by growth factors and inflammatory cytokines (e.g., transforming growth factor-β [TGF-β], tumor necrosis factor-α [TNF-α], interleukin [IL]-1, IL-8) released in response to talc-induced inflammation of the tissues lining the thoracic cavity as part of the repair process [2–4]. Imaizumi et al. found that significantly higher levels of vascular endothelial growth factor and TGF-β were present in the pleural fluid of patients for whom pleurodesis was successful vs unsuccessful [7]. The anti-inflammatory actions of steroids include inhibition of NF-κB activation and reduced expression of IL-1, IL-6, and other inflammatory cytokines; accordingly, steroid use has been associated with a reduction in the efficacy of talc pleurodesis [8]. However, in the present study, we did not detect an effect of SST on the success of pleurodesis among patients in good general condition.

Pleurodesis using a total of > 10 g talc is reportedly associated with an increased incidence of ARDS and other acute respiratory events [9]. Given that the usual amount of talc per procedure is 4 g, this risk should only arise when pleurodesis is performed more than twice. Our patients underwent a maximum of two procedures, and none suffered from ARDS or any other acute respiratory events. Steroids are used to treat ARDS. Therefore the patients who are receiving steroids may be less likely to develop ARDS.

The main limitations of this study were its retrospective nature and its small sample size; as such, our data cannot be used to draw firm conclusions. Collection of data on additional cases should clarify the precise relationship between steroid dose and pleurodesis efficacy, as well as the doses at which tapering should be performed before SST cessation. If patients must undergo pleurodesis while on SST and a delay is acceptable*,* it may be preferable to perform the procedure when the effusion volume has decreased.

In conclusion, our study suggests that physicians should carefully weigh the feasibility of continuing SST in patients before and after talc pleurodesis on a case-by-case basis.

## Data Availability

Data used in this case report will not be shared due to the risk of identifying the patients’ identities. However, most of the relevant data are presented here.

## Abbreviations

ARDS: Acute respiratory distress syndrome
mPSL: methylprednisolone
PSL: Prednisolone
SST: Systemic steroid therapy
